# Diagnostic and Predictive Values of Ferroptosis-Related Genes in Child Sepsis

**DOI:** 10.3389/fimmu.2022.881914

**Published:** 2022-06-30

**Authors:** Zhi Li, Chi Zhang, Yiqi Liu, Fang Wang, Baoling Zhao, Junmei Yang, Yongjing Zhao, Hong Zhao, Guiqiang Wang

**Affiliations:** ^1^ Department of Infectious Diseases, Children’s Hospital Affiliated to Zhengzhou University, Zhengzhou, China; ^2^ Department of Infectious Disease, Center for Liver Disease, Peking University First Hospital, Beijing, China; ^3^ Department of Laboratory Medicine, Children’s Hospital Affiliated to Zhengzhou University, Zhengzhou, China; ^4^ Department of Infectious Diseases, Peking University International Hospital, Beijing, China

**Keywords:** sepsis, children, MAPK14, ferroptosis, immune landscape

## Abstract

**Background:**

Early diagnosis of sepsis in children was essential to reducing mortality. This study aimed to explore the value of ferroptosis-related genes in children with sepsis.

**Methods:**

We screened the septic children microarray dataset from the GEO database and analyzed the ferroptosis-related differentially expressed genes (DEGs). A functional analysis of ferroptosis-related DEGs was performed. The protein–protein interaction network was used to identify hub genes. We explored the immune landscape of sepsis and controls. The value of hub genes in diagnosing sepsis was tested in the training (GSE26440) and validation sets (GSE13904), and ELISA was used to verify their diagnostic value in children with sepsis in our hospital.

**Results:**

A total of 2,103 DEGs in GSE26440 were obtained, of which ferroptosis-related DEGs were 34. Enrichment analysis showed significant enrichment in the ferroptosis and hypoxia pathways (i.e., HIF-1 pathway). The top three genes (HMOX1, MAPK14, TLR4) were selected as hub genes. Immunological analysis suggested that 10 cell types (i.e., CD8/CD4 T cells) were lower in sepsis. Immune checkpoint-related genes CD274 (PD-L1), HAVCR2 (TIM3), and SIGLEC15 were overexpressed in sepsis. The AUROC for the diagnosis of sepsis for HMOX1 and TLR4 ranged from 0.77 to 0.81, while the AUROC of MAPK14 reached 0.935 and 0.941 in the training and validation sets. Serum ELISA results of HMOX1 and TLR4 showed no significant difference in differentiating sepsis. The AUROC of MAPK14 was 0.877. When the diagnostic threshold was 74.852 ng/ml, the sensitivity and specificity were 0.906 and 0.719, respectively.

**Conclusion:**

Ferroptosis-related gene MAPK14 is of considerable value in the early diagnosis of sepsis in children.

## Introduction

Sepsis is a life-threatening organ dysfunction mainly caused by the maladjusted response of the host to infection (1). Sepsis is a major public health problem that affects millions of people worldwide each year, with a death rate of between one-third and one-sixth (2). It was estimated that 20.3 million children under 5 years of age suffer from sepsis worldwide, resulting in 2.9 million global deaths (3, 4). Early diagnosis and timely effective intervention can significantly reduce the mortality (5). Shi et al.’s study observed that the expression of GPX4 in vascular endothelial cells of septic rats decreased significantly. Lipid peroxidation in vascular endothelial cells stimulated by LPS was also significantly enhanced. The structures of pulmonary vein mitochondria in septic rats presented the hallmarks of ferroptosis (i.e., fragmented morphology with loss of cristae), observed with transmission electron microscopy. Their further experiments showed that dexmedetomidine could alleviate sepsis-induced vascular leakage through reprogramming the pentose phosphate pathway (*via* activation of the Nrf2/G6PD pathway) (6). Xiao et al. showed that the cardiac systolic function of septic rats decreased significantly, compared with the control group. However, this decrease could be alleviated by ferrostatin-1 (a ferroptosis inhibitor) and reduce the death rate of septic rats. Subsequent experiments suggested that this may be related to the inhibition of the TLR4/NF-κB signaling pathway (7). In addition, Wang et al.’s research also supports the conclusion of dexmedetomidine-alleviated sepsis−induced myocardial ferroptosis and septic heart injury (8). In brief, ferroptosis may play an important role in the occurrence and development of sepsis.

Ferroptosis, first proposed by Brent R. Stockwell of Columbia University in 2012, is an iron-dependent, novel form of programmed cell death that is distinct from apoptosis, cell necrosis, and cell autophagy (9, 10). Previous studies related to ferroptosis have focused on various malignancies (11). Recently, an increasing number of studies have shown that ferroptosis also plays a considerable role in various non-neoplastic diseases, such as Alzheimer’s disease (12), Parkinson’s disease (13), and renal failure (14).

Besides the diseases mentioned above, ferroptosis also plays a significant role in sepsis. We searched for the relationship between sepsis and ferroptosis in PubMed using the following search formula: (“ferroptosis” [Title/Abstract]) AND (“sepsis”[Title/Abstract] OR “pyemia”[Title/Abstract] OR “septicemia”[Title/Abstract] OR “pyohemia”[Title/Abstract]). A total of 21 relevant studies were retrieved, most of which were about the role of ferroptosis in the pathophysiological process of sepsis and shock (6, 15, 16). Early diagnosis of sepsis is crucial to reducing mortality (17). However, few studies have focused on the value of ferroptosis-related genes in the early diagnosis of sepsis, let alone in childhood sepsis. Based on this, we explored the value of ferroptosis-associated genes in early diagnosis by searching the Gene Expression Omnibus (GEO, http://www.ncbi.nlm.nih.gov/geo) and validated our findings using samples from children with sepsis in our hospital.

## Materials and Methods

### Microarray Dataset Collection and Data Process

Microarray datasets were screened from GEO. The search keywords were “sepsis” and “children,” with the following searching strategies: ((“sepsis”[MeSH Terms] OR sepsis[All Fields]) AND (“child”[MeSH Terms] OR children[All Fields])) AND “Homo sapiens”[porgn] AND (“gse”[Filter] AND “Expression profiling by array”[Filter]). Finally, GSE26440 (as training set) and GSE13904 (as validation set) were obtained. GSE26440 contained 130 whole blood-derived RNA samples from 98 children with septic shock and 32 normal controls. GSE13904 contained 158 children with sepsis and 18 normal controls. This study was approved by the Ethics Committee of Children’s Hospital Affiliated to Zhengzhou University (2021-H-K21).

### Screening Ferroptosis-Related Differentially Expressed Genes

We transformed the probe into gene symbol in each dataset based on the platform’s annotation file, when there were multiple probes mapped to the same gene symbol; the mean value of probes was selected as the gene expression value. Differentially expressed genes (DEGs) between sepsis and control were analyzed *via* the “limma package” in R software, with the following cutoff for adjustment: p value < 0.05 and FC (fold changes) > 1.5. Collection and collation of ferroptosis-related genes from the “FerrDb” database were performed (18). The intersection of DEGs and ferroptosis-related genes was visualized by the Venn plot.

### Functional Enrichment Analysis and Protein–Protein Interaction Network Analysis of Ferroptosis-Related DEGs

We used the “clusterProfiler” package (19) of R to perform the Gene Ontology (GO) and Kyoto Encyclopedia of Genes and Genomes (KEGG) enrichment analyses of ferroptosis-related DEGs. GO analysis included three categories, biological process (BP), cellular component (CC), and molecular function (MF), which was important in the exploration of biological functions (20). KEGG analysis was used to explore potential pathways (21).

The protein–protein interaction (PPI) network analysis of ferroptosis-related DEGs was conducted using the STRING online website (https://string-db.org/). The results of STRING were then imported into Cytoscape (version 3.8.2), and the key subnetworks were extracted using the cytoHubba plugin. Finally, the three genes with the highest scores (maximum correlation criterion, MCC algorithm) in the subnetworks were selected as hub genes.

### Landscape of Immune Infiltration and Immune Checkpoint Genes Between Sepsis and Controls

The development of sepsis was closely related to our immune system against pathogenic microorganisms (22). Therefore, we used the CIBERSORT tool (23) to explore the difference in immune cell marker expression between sepsis and controls. CIBERSORT is a tool for deconvoluting the expression matrix of human immune cell subtypes in the principle of linear support vector regression. The method was based on a known reference set that provides a set of gene expression features for 22 immune cell subtypes. What is more, we also analyzed the differences in the expression of immune checkpoint genes between sepsis and control groups.

### Enzyme-Linked Immunosorbent Assay

Serum HMOX1 (Ruixin Biotech, Zhejiang, China), MAPK14 (Jianglai Biological Technology, Shanghai, China), TLR4 (Cloud-Clone Corp., Wuhan, China), GPX4 (Cloud-Clone Corp., China), and TFRC (Cloud-Clone Corp., China) levels were determined using the human enzyme-linked immunosorbent assay (ELISA) kits according to the manufacturer’s instructions, following the steps as follows: step 1: adding the standard working solution and the sample into the hole in turn, with each hole 100 μl (then, incubating at 37°C for 90 min); step 2: discarding the liquid and adding a biotinylated antibody/antigen working solution 100 μl (then, incubating at 37°C for 60 min); step 3: throwing out the liquid and adding 350 μl washing solution to each hole, soaking for 1–2 min, and shaking off the liquid (with absorbent paper), repeating this washing step three times; step 4: adding the enzyme conjugate working solution and incubating at 37°C for 30 min; step 5: washing the plate for five times (same as step 3); step 6: adding the chromogenic solution for color development, and terminating the color development; and step 7: measuring the optical density of each hole by enzyme labeling instrument at 450-nm wavelength.

### Statistical Analysis

Student’s t-test or Kruskal–Wallis H test was used to compare continuous variables; chi-square test or Fisher’s exact test was used for categorical variables. The diagnostic accuracy of three hub genes was analyzed with receiver operating characteristic curves (ROC) and expressed as the area under the ROC curves (AUROC) and 95% CI. The sensitivity, specificity, positive predictive value (PPV), negative predictive value (NPV), positive likelihood ratio (PLR), and negative likelihood ratio (NLR) for each gene were calculated. The optimal cutoff values of hub genes were obtained when Youden’s index was fixed at the maximum value. Spearman’s rank tests or Pearson correlation coefficient were used to analyze the associations between hub genes and immune cells and immune checkpoint genes. All statistical analyses were performed using R software (version 4.1.0) and SPSS version 19.0 software (SPSS, Inc., Chicago, IL, USA). P values less than 0.05 (two-sided) were considered statistically significant.

## Results

### Identification of Ferroptosis-Related DEGs Between Sepsis and Control

**Figure 1:**
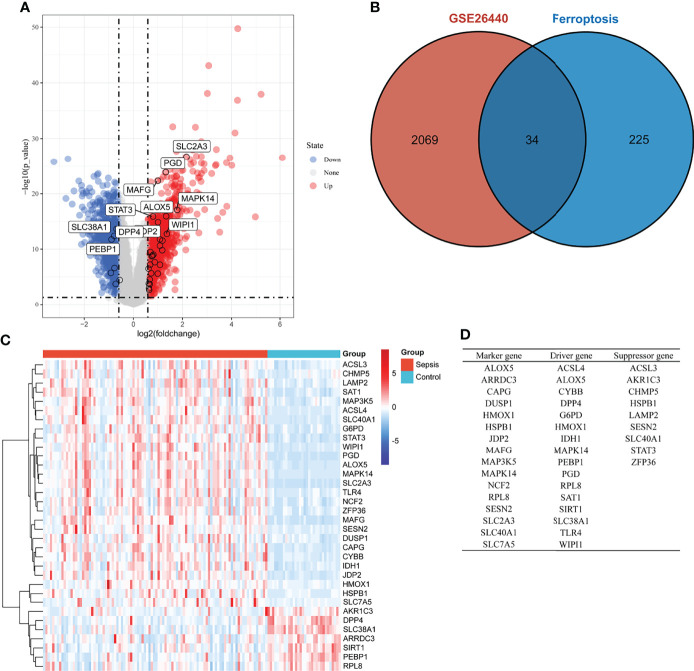
Overview of the differentially expressed ferroptosis genes in children with sepsis and controls. **(A)** Volcano plot of genes differentially expressed between sepsis children and controls in the GSE26440 dataset. Blue nodes represent down-regulation in sepsis; red nodes represent up-regulation; and gray nodes represent no significant difference from controls. **(B)** Intersection of differentially expressed genes (DEGs) in the GSE26440 and ferroptosis genes. The count on the left (2,069 genes) refers to DEGs unique to GSE26440; the count in the middle (34 genes) refers to ferroptosis-related DEGs; and the count on the right (225 genes) refers to unique to ferroptosis genes. **(C)** Heat map of 34 ferroptosis-related DEGs. **(D)** Classification of 34 ferroptosis-related DEGs. Of these, 16 are marker genes, 16 are driver genes, and 9 are suppressor genes. ALOX5, MAPK14, and RPL8 may be both marker and driver genes; HSPB1, SESN2, and SLC40A1 may be both marker and suppressor genes; HMOX1 may be both marker, driver, and suppressor genes.

DEGs in whole blood-derived RNA samples from sepsis children and normal controls were analyzed using the “limma” package. In total, 2,103 DEGs were identified by screening, of which 1,103 were downregulated (**Figure 1A**, blue dot) and 1,000 were upregulated genes (**Figure 1A**, red dot). Then, we extracted ferroptosis-related genes from the “FerrDb” database, as shown in **Figure 1B**; the count on the left (2,069 genes) refers to DEGs unique to GSE26440; the count in the middle (34 genes) refers to ferroptosis-related DEGs; and the count on the right (225 genes) refers to unique ferroptosis genes. The heat map in **Figure 1C** shows the standardized expression of ferroptosis-related DEGs (27 up-regulated and 7 down-regulated). **Figure 1D** shows the classification of 34 ferroptosis-related DEGs. Of these, 16 were marker genes, 16 were driver genes, and 9 were suppressor genes. ALOX5, MAPK14, and RPL8 may be both marker and driver genes; HSPB1, SESN2, and SLC40A1 may be both marker and suppressor genes; HMOX1 may be both marker, driver, and suppressor genes.

### Enrichment Analysis of Ferroptosis-Related DEGs and PPI Network Analysis

**Figure 2:**
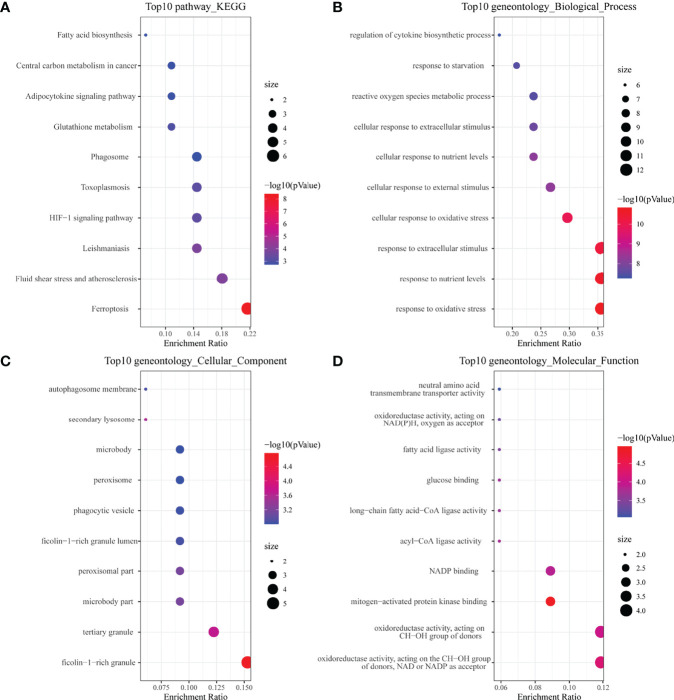
Enrichment analysis of ferroptosis-related DEGs. **(A)** Top 10 KEGG pathway. **(B)** Top 10 GO (gene ontology) biological processes pathway. **(C)** Top 10 GO cellular component pathway. **(D)** Top 10 GO molecular function pathway.

In the KEGG analysis (**Figure 2A**), the top 10 enriched pathways were mainly ferroptosis, HIF-1 signaling pathway, glutathione metabolism, etc. In GO-BP analysis (**Figure 2B**), the major pathways were also hypoxia or oxidative stress. In GO-CC analysis (**Figure 2C**), a ficolin-1-rich granule was significantly enriched. Ficolin is a considerable initiator in the complement agglutination pathway, which could bind to glycans on the surface of microorganisms and may play a momentous role in anti-infective immunity. The results of enrichment analysis in GO-MF (**Figure 2D**) were also mainly to the activation of oxidative stress-related pathways. The results of the enrichment analysis suggested that oxidative stress and activation of the complement pathway may play an important role in childhood sepsis.

**Figure 3:**
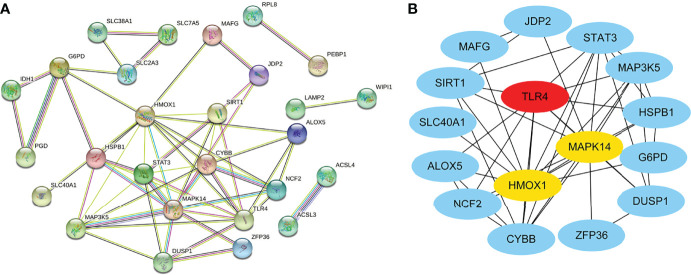
Protein–protein interaction (PPI) network of ferroptosis DEGs. **(A)** PPI network of differentially expressed ferroptosis DEGs. **(B)** Subnetwork of hub genes from the PPI network. Node color reflects the degree of connectivity (red color represents a higher degree, and blue color represents a lower degree).

We obtained the PPI network (**Figure 3A**) containing 34 nodes and 56 edges. Seven of the 34 genes were not related to other molecules and did not form a molecular network. The network was set to the default cutoff (interaction score > 0.4) in the STRING online database. The Cytoscape CytoHubba plug-in was used to identify hub genes. The top three genes (HMOX1, MAPK14, TLR4) with the highest scores (MCC algorithm) were selected as hub genes (**Figure 3B**).

### Landscape of Immune Infiltration and Immune Checkpoint Genes Between Sepsis and Controls

**Figure 4:**
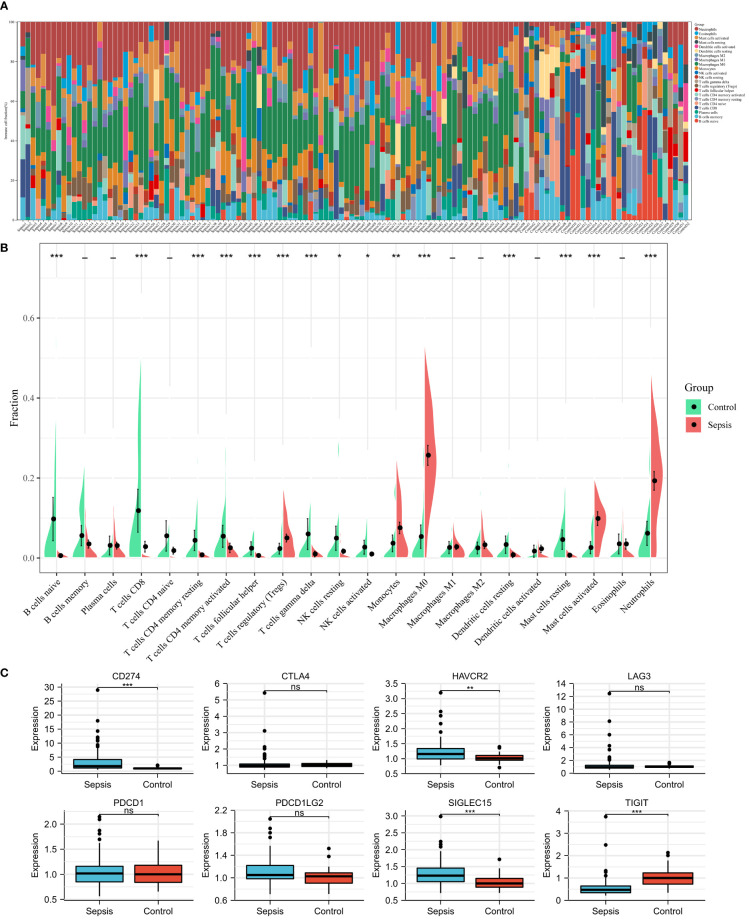
Landscape of immune infiltration and immune checkpoint genes between sepsis and controls in children in the GSE26440 dataset. **(A)** Bar charts of 22 immune cell proportions in sepsis and controls. The first 98 were sepsis, and the last 32 were controls. **(B)** Differential expression of different types of immune cell marker expression between sepsis and controls. **(C)** Differential expression of different immune checkpoint genes between sepsis and controls. *P < 0.05, **P < 0.01, ***P < 0.001, ns P > 0.05.

Using the CIBERSORT algorithm, we analyzed the differences in peripheral blood immune cells between the sepsis and control groups. **Figure 4A** shows the expression of a proportion of 22 immune cell markers in all patients. The first 98 comprised the sepsis group, and the last 32 comprised the control group. As shown in **Figure 4B**, the expressions of 15 types of immune cell markers were significantly different. Five types of immune cell markers (T cells regulatory (Tregs), monocytes, macrophages M0, mast cells activated, and neutrophils) have a significantly higher expression in the sepsis group. Meanwhile, 10 types of immune cell markers (B cells naive, T cells CD8, T cells CD4 memory resting, T cells CD4 memory activated, T cells follicular helper, T cells gamma delta, NK cells resting, NK cells activated, dendritic cells resting, and mast cells resting) have a lower expression compared with controls.

Next, using microarray data, we analyzed the difference in immune checkpoint gene expression between sepsis and control. There were significant differences in four of the eight immune checkpoint genes (**Figure 4C**), including three high-expression (CD274 [coding programmed death ligand 1, PD-L1], HAVCR2 [coding T cell immunoglobulin mucin 3, TIM3], and SIGLEC15 [coding sialic acid binding Ig-like lectin 15, SIGLEC-15]) and one-low expression (TIGIT [coding T cell immunoreceptor with Ig and ITIM domains]), compared with the control group. In short, immune checkpoint genes were highly expressed in sepsis.

### Correlation of HMOX1, MAPK14, and TLR4 With Immune Cells and Immune Checkpoint Genes

**Figure 5:**
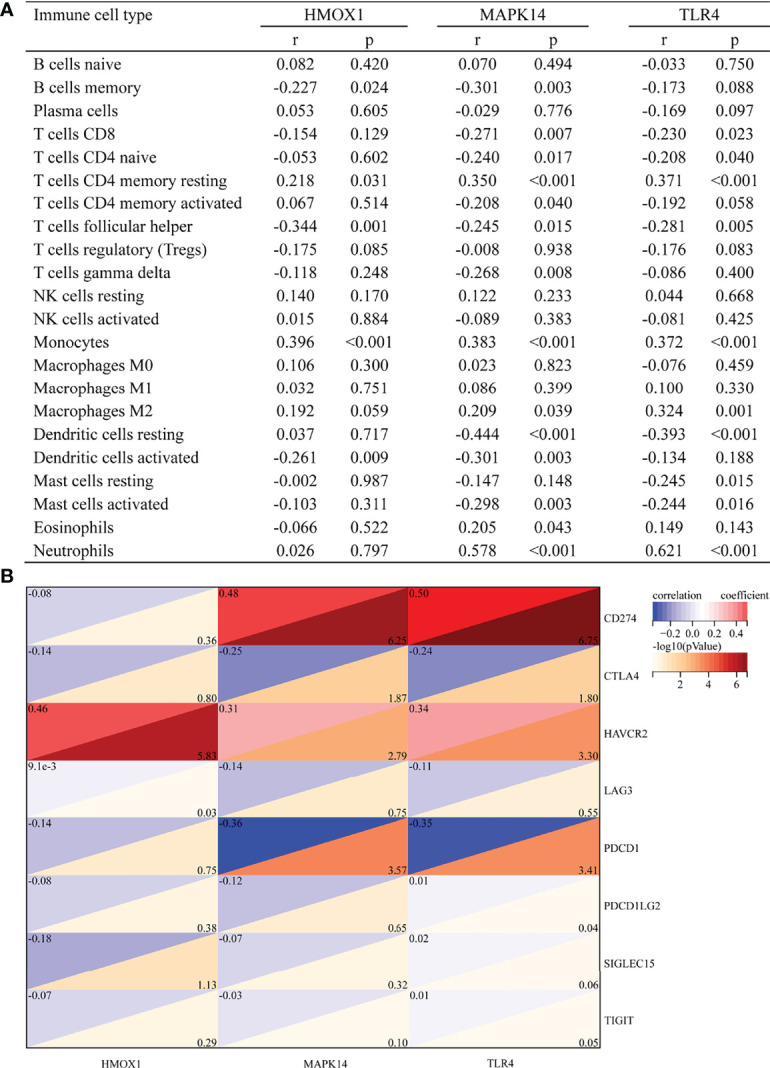
Correlation between hub gene and immune cells and immune checkpoint genes. **(A)** Correlation between hub gene and immune cells. **(B)** Correlation between hub gene and immune checkpoint genes.

**Figure 5A** shows the correlation between three hub genes and immune cells. HMOX1 was significantly correlated with five immune cells, among which monocytes had the strongest correlation (r = 0.396, P < 0.001). MAPK14 was significantly correlated with 14 immune cells, and the strongest correlation was that of neutrophils (r = 0.578, P < 0.001), most of which were negatively correlated (9/14), except T cells CD4 memory resting, monocytes, macrophages M2, eosinophils, and neutrophils. TLR4 was significantly positively correlated with four immune cells and negatively correlated with six immune cells. The strongest correlation was that of neutrophils (r = 0.621, P < 0.001). In addition, all three hub genes belonged to the driver genes of ferroptosis (**Figure 1D**).

The correlation with immune checkpoints is shown in **Figure 5B**. HMOX1 was only significantly associated with HAVCR2 (r = 0.46, P < 0.001). MAPK14 was significantly positively correlated with CD274 (r = 0.48, P < 0.001) and HAVCR2 (r = 0.31, P = 0.002) but negatively correlated with CTLA4 (r = -0.25, P = 0.013) and PDCD1 (r = -0.36, P < 0.001). TLR4 was positively correlated with CD274 (r = 0.5, P < 0.001) and HAVCR2 (r = 0.34, P = 0.001) and negatively correlated with CTLA4 (r = -0.24, P = 0.016) and PDCD1 (r = -0.35, P < 0.001). In brief, HAVCR2 was significantly positively correlated with the three hub genes. MAPK14 and TLR4 was positively correlated with CD274, while it was negatively correlated with PDCD1.

### Performance of Hub Genes to Diagnose Sepsis in the Training Set

**Figure 6:**
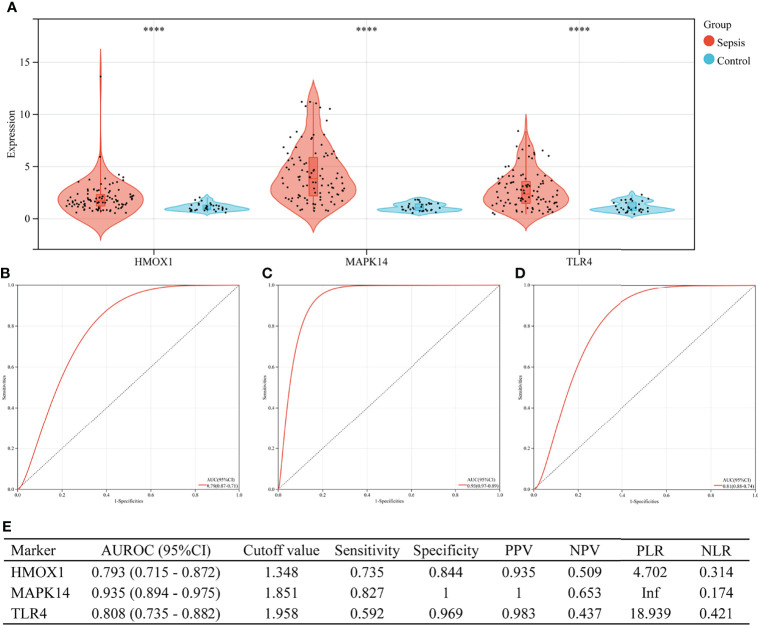
The performance of hub genes to diagnose sepsis in the GSE26440 training set. **(A)** Expression difference of three hub genes in the sepsis and control groups. **(B)** The receiver operating characteristic (ROC) curve of HMOX1 gene diagnosis of sepsis. **(C)** The ROC curve of MAPK14 gene diagnosis of sepsis. **(D)** The ROC curve of TLR4 gene diagnosis of sepsis. **(E)** Diagnostic value of three hub genes for differentiating between sepsis and control groups. PPV, positive predictive value; NPV, negative predictive value; PLR, positive likelihood ratio; NLR, negative likelihood ratio; AUROC, area under the receiver operating characteristics curve; CI, confidence interval; ****P < 0.0001.

**Figure 6** shows the value of three ferroptosis-related hub genes in the diagnosis of sepsis in the training set (GSE26440). HMOX1, MAPK14, and TLR4 were significantly overexpressed in sepsis, compared with the control group (all P < 0.001, **Figure 6A**). The area under the ROC curve (AUROC) of HMOX1 in the diagnosis of sepsis was 0.793 (95% CI 0.715–0.872), with the sensitivity and specificity of 0.735 and 0.844, respectively (**Figures 6B, E**). The AUROC of MAPK14 in the diagnosis of sepsis was 0.935 (95% CI 0.894–0.975), and the corresponding sensitivity and specificity were 0.827 and 1, respectively. Moreover, the PPV, NPV, and NLR were 1, 0.653, and 0.174, respectively. Since the number of false positives was 0, the PLR cannot be calculated (**Figures 6C, E**). The AUROC of TLR4 in the diagnosis of sepsis was 0.808 (95% CI 0.735–0.882), with the sensitivity and specificity of 0.592 and 0.969, respectively (**Figures 6D, E**).

### Performance of Hub Genes to Diagnose Sepsis in the Validation Set

**Figure 7:**
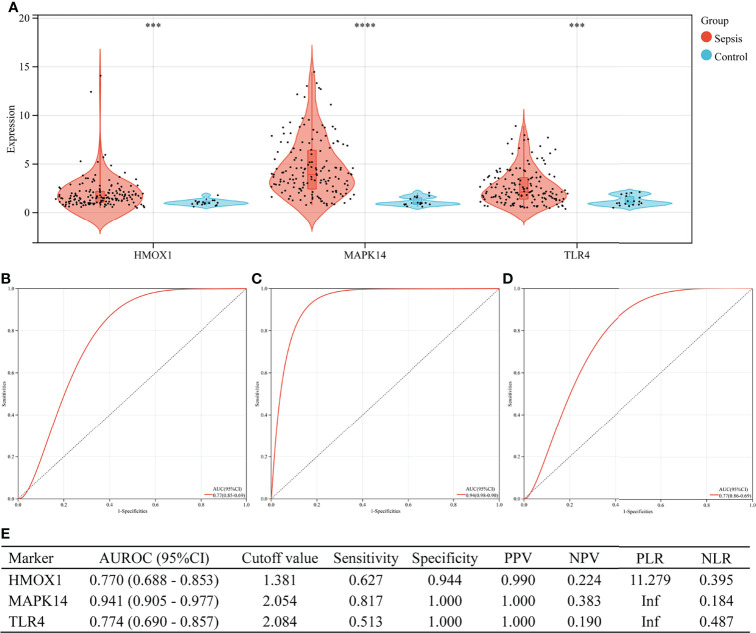
The performance of hub genes to diagnose sepsis in the GSE13904 validation set. **(A)** Expression difference of three hub genes in sepsis and control groups. **(B)** Receiver operating characteristic (ROC) curve of HMOX1 gene diagnosis of sepsis. **(C)** ROC curve of MAPK14 gene diagnosis of sepsis. **(D)** ROC curve of TLR4 gene diagnosis of sepsis. **(E)** Diagnostic value of three hub genes for differentiating between sepsis and control groups. PPV, positive predictive value; NPV, negative predictive value; PLR, positive likelihood ratio; NLR, negative likelihood ratio; AUROC, area under the receiver operating characteristics curve; CI, confidence interval; ***P < 0.001, ****P < 0.0001.

In the validation set (GSE13904), the three hub genes were also excellent in the diagnosis of sepsis (**Figure 7**). There were 176 children in the validation group, including 158 cases of sepsis. **Figure 7A** shows the expressions of HMOX1, MAPK14, and TLR4 in the sepsis and control groups (all P < 0.001). The AUROC of HMOX1 (**Figure 7B**), MAPK14 (**Figure 7C**), and TLR4 (**Figure 7D**) in the diagnosis of sepsis were 0.770, 0.941, and 0.774 respectively. The specificity and PPV were all more than 0.9 (**Figure 7E**).

### Verification of Serum HMOX1, MAPK14, and TLR4 Concentrations in the External Validation Set

**Figure 8:**
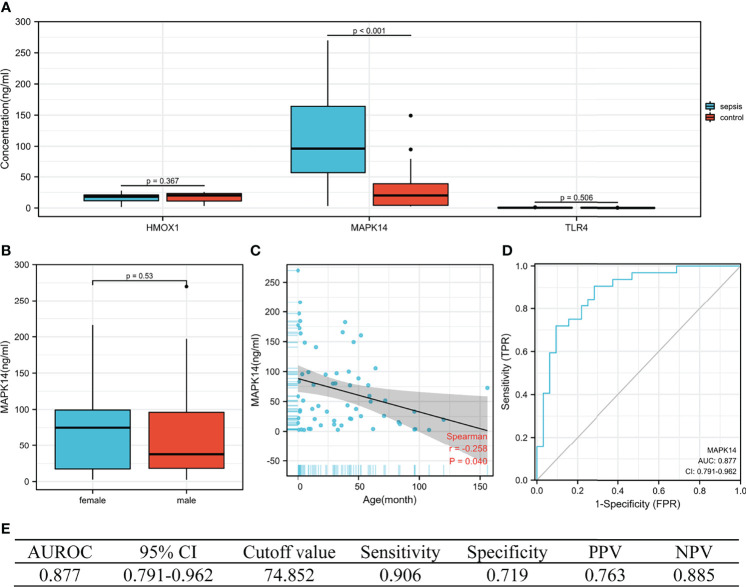
The performance of hub genes in the validation cohort. **(A)** Concentration of three hub proteins in serum. **(B)** Concentration of MAPK14 in different genders. **(C)** Correlation between MAPK14 and age. **(D)** ROC curve of serum MAPK14 protein diagnosis of sepsis. **(E)** Diagnostic value of serum MAPK14 for differentiating between sepsis and control patients. PPV, positive predictive value; NPV, negative predictive value; AUROC, area under the receiver operating characteristics curve; CI, confidence interval.

The concentration of the three proteins in serum between sepsis and control was validated (by ELISA). We collected blood samples from 32 children with sepsis and 32 children with respiratory tract infection in our hospital, of which 68.8% (44/64) were men, with a median age of 30.0 months (IQR 3.9–52.0), from October to December 2021. As shown in **Figure 8A**, MAPK14 concentrations were significantly higher in children with sepsis than in controls (median 96.418 vs. 20.177 ng/ml, P < 0.001), whereas HMOX1 (P = 0.367) and TLR4 (P = 0.506) were not significantly different. Subsequently, our analysis revealed that MAPK14 in serum was independent of gender (P = 0.530, **Figure 8B**) and significantly negatively correlated with age (r = -0.258, P = 0.040, **Figure 8C**). The diagnostic test showed that serum MAPK14 had excellent performance in diagnosing sepsis, with AUROC of 0.877 (**Figure 8D**). When the diagnostic threshold was 74.852 ng/ml, the sensitivity and specificity were 0.906 and 0.719, respectively; the PPV and NPV were 0.763 and 0.885, respectively (**Figure 8E**).

### Difference in GPX4 and TFRC in Sepsis and Control

**Figure 9:**
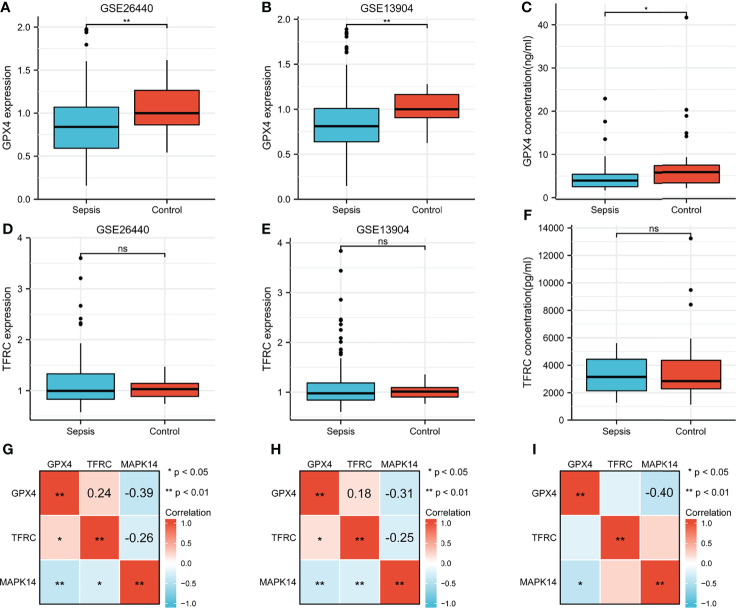
Difference of GPX4 and TFRC in sepsis and control. GPX4 expression between sepsis and control in **(A)** GSE26440 cohort and **(B)** GSE13904 cohort. **(C)** Serum GPX4 concentration between sepsis and control in our ELISA result. TFRC expression between sepsis and control in the **(D)** GSE26440 cohort and **(E)** GSE13904 cohort. **(F)** Serum TFRC concentration between sepsis and control in our ELISA result. Correlation analysis between GPX4, TFRC, and MAPK14 in sepsis in the **(G)** GSE26440 cohort and **(H)** GSE13904 cohort and **(I)** our ELISA result. *P < 0.05; **P < 0.01; "ns", not significant.

GPX4 and TFRC played an important role in ferroptosis, so it was necessary to analyze the difference between sepsis and control. GPX4 was significantly overexpressed in the GSE26440 cohort (**Figure 9A**), validation cohort GSE13904 (**Figure 9B**), and serum ELISA results (**Figure 9C**). However, there was no significant difference in TFRC (**Figures 9D–F**). The correlation analysis between GPX4, TFRC, and MAPK14 is shown in **Figures 9G–I**. Whether it was in the GSE26440 cohort, in the GSE13904 validation cohort, or in our serum ELISA result, MAPK14 was significantly negatively correlated with GPX4, and the correlation coefficients were -0.39 (**Figure 9G**), -0.31 (**Figure 9H**), and -0.4 (**Figure 9I**), respectively.

## Discussion

Sepsis could cause life-threatening organ dysfunction both in adults and in children (1, 17). More and more studies have shown that ferroptosis played an unignored role in sepsis (24). The present study analyzed different ferroptosis-related DEGs in sepsis and healthy controls and explored the clinical value of three hub genes (HMOX1, MAPK14, TLR4) in the early diagnosis of sepsis. The imbalance of human immune function was inextricably related to sepsis (25). Then, we used the CIBERSORT algorithm to analyze the differences in peripheral blood immune cells and immune checkpoint gene expression between sepsis and control groups and analyze the correlation with hub genes. Finally, we explored the value of hub genes in the diagnosis of sepsis in the training set and validation set and verified them in serum of septic children by ELISA in our hospital.

Immune dysregulations in sepsis were self-evident, whether innate immunity or adaptive immunity (25). Our results showed that innate immune cells including monocytes, macrophages M0, mast cells activated, and neutrophils were significantly up-regulated in children with sepsis. Asmaa et al.’s study of 30 neonatal sepsis (a total of 60 participates) showed that compared with the control group, CD86 + monocytes in the sepsis group were significantly higher (median 78.4% vs. 24.0%, P < 0.001). When the cutoff value was 36%, the AUROC for early diagnosis of neonatal sepsis was as high as 0.951, and the sensitivity and specificity were 90% (26). Céspedes et al.’s malaria-associated bacteremia in a rodent model demonstrated a significantly high expression of mast cell-activating cytokines (IL-9 and IL-13). This was followed by a concomitant increase in circulating IgE, which can induce mast cell degranulation, and mast cell protease 1 (Mcpt-1) and Mcpt-4 were observed concurrently with bacteremia and increased intestinal permeability. Immunohistochemistry also showed a significant increase in mast cells in ileal tissue 4 days after the appearance of bacteremia compared to controls (27).

With regard to adaptive immunity, our analysis indicated that CD4+ T cells, CD8+ T cells, T cells follicular helper, and B cells were all down-regulated in patients with sepsis, except regulatory T cells regulatory (Tregs). The low expression of CD8+ T cells in patients with sepsis has been proved by many studies (22, 28). It was worth noting that Jensen et al.’s recent research showed that sepsis may lead to continuous changes in the phenotype and function of CD8 T cells. The sepsis-induced changes in the composition of the memory CD8 T-cell pool and transcriptional landscape culminated in an altered T-cell function and reduced capacity to control *L. monocytogenes* infection. This may change the host’s capacity to respond to reinfection (29). In a prospective cohort study, B cells and follicular T cells were linked. A higher proportion of mature B cells and circulating follicular T (cTfh) cells were found in survivors compared with non-survivors (sepsis onset: memory B cells: 3.44% vs. 4.48%, antibody-secreting cells: 4.53% vs. 6.30%, cTfh cells: 3.57% vs. 4.49%; 24 h after sepsis onset: memory B cells: 4.05% vs. 7.20%, antibody-secreting cells: 5.25% vs. 8.78%, cTfh cells: 3.98% vs. 6.15%). Moreover, the numbers of cTfh cell positively correlated with the numbers of memory B cells (sepsis onset: r = 0.48, P < 0.001; 24 h after sepsis onset: r = 0.54, P < 0.001) (30).

Our analysis showed that multiple immune checkpoints (CD274 [coding PD-L1], HAVCR2 [coding TIM3], and SIGLEC15 [coding SIGLEC-15]) were also highly expressed in sepsis. Wang et al.’s research indicated that an increased PD-L1 expression on human neutrophils delays cellular apoptosis by triggering PI3K-dependent AKT phosphorylation to drive lung injury and increase mortality during clinical and experimental sepsis (31). In addition, there was also a clinical trial of PD-L1 monoclonal antibody in the treatment of sepsis (a phase 1b/2a, randomized, double-blinded, placebo-controlled, multicenter study to evaluate the safety, tolerability, pharmacokinetics, and pharmacodynamics of BMS-936559 in subjects with severe sepsis; NCT02576457) (32).

Ferroptosis is an iron-related death where the ferroportin iron exporter played an essential role. However, SLC40A1, encoding ferroportin-1, was upregulated in our analysis (**Figure 1C**). Theoretically, when ferroptosis occurs in septic patients, SLC40A1-mediated iron export was inhibited (33). However, SLC40A1 should be increased from a clinical or homeostasis view. The reasons were as follows: first, patients with sepsis present with elevated ferritin and serum iron clinically, which was a manifestation of iron aggregation in a patient’s body (1). Second, anemia was not rare in sepsis patients, where an important cause of anemia was hemolytic anemia due to red blood cell rupture, especially in the event of shock or DIC (34). Third, the treatment of patients with infectious shock will be transfused with blood, either plasma or concentrated red blood cells, which will increase the body’s iron accumulation. All the mentioned causes of iron accumulation in patients were acutely increased, not slow increasing. In order to maintain the homeostasis, iron transport was bound to increase as well. Therefore, the SLC40A1-mediated iron export increase would not be unusual. In brief, we speculated that iron accumulation in septic patients was primarily a source of increased iron, with a decreased iron output as a secondary cause. This may distinguish the occurrence of ferroptosis in patients with chronic diseases or neoplasms (11).

We obtained three ferroptosis-related DEGs as key genes based on the aforementioned analysis. The AUROC of three genes in the early diagnosis of sepsis was remarkable in both training and validation sets. Studies have shown that in sepsis patients, HMOX1 expression was higher in non-survivors than in survivors (35). Other relevant animal experiments also support this result (36, 37). Ding et al.’s animal experiments revealed that the expression of MAPK14 in septic mice was significantly higher than that in healthy control mice. Moreover, miR-128-3p can enhance the protective effect of dexmedetomidine on acute lung injury in septic mice by inhibiting the expression of MAPK14 (38). In addition to TLR4 as a marker of early diagnosis of sepsis, Maiti et al. found that lumican (an extracellular matrix protein) promoted TLR4 response to bacterial lipopolysaccharides but restricts nucleic acid-specific TLR9 in macrophages and dendritic cells. Moreover, endocytosed lumican colocalizes with TLR4 and LPS and promotes endosomal induction of type I interferons, in explaining the effects of the extracellular matrix on infection and immunity (39). However, our results did not support HMXO1 and TLR4. We speculate that this may be due to the following reasons. Firstly, we checked the problems related to the experimental operation. The R^2^ values of the two standard curves were 0.9957 and 0.9979, which excluded the experimental reasons. Subsequently, we reckoned this possible association with very low concentrations in peripheral blood. Finally, it is because the children included in this study were all young, with 34.4% of patients less than 1 year old, and studies on HMXO1 and TLR4 in infants have not been reported. In short, there was an eminent value of MAPK14 in the diagnosis of sepsis in children, while HMXO1 and TLR4 still need to be further explored in children.

Our research also inevitably has some limitations. First, although we used the peripheral blood of children in our center to verify the diagnostic value of the above three genes, there was a lack of signal pathway-related mechanisms for further verification. Second, the proportion of immune cells was extrapolated using the CIBERSORT deconvolution algorithm rather than true tests of the number in peripheral blood, which may have some discrepancies with the authentic results. Third, the population in this study was children, and the diagnostic value of MAPK14 in adults with sepsis needs further validation.

## Conclusion

This study showed that ferroptosis-related gene MAPK14 was of prominent value in the early diagnosis of sepsis in children. We also provided insight into the landscape of immune cells and the expression of immune checkpoints in children underlying sepsis. However, future studies of ferroptosis in children with sepsis need to be further meticulously explored.

## Data Availability Statement

The datasets presented in this study can be found in online repositories. The names of the repository/repositories and accession number(s) can be found in the article/supplementary material.

## Ethics Statement

The studies involving human participants were reviewed and approved by the Ethics Committee of Children’s Hospital Affiliated to Zhengzhou University (2021-H-K21). Written informed consent from the participants’ legal guardian/next of kin was not required to participate in this study in accordance with the national legislation and the institutional requirements.

## Author Contributions

ZL and CZ drafted the manuscript. CZ and YL participated in the bioinformatics analysis. ZL, FW, BZ, JY, and YZ participated in the serum sample collection. HZ and GW provided the overall principle and direction of the study. All authors contributed to the article and approved the submitted version.

## Funding

This study was supported by the Project of Beijing Science and Technology Committee (Z191100007619037); China Mega‐Project for Infectious Diseases (2017ZX10203202, 2013ZX10002005).

## Conflict of Interest

The authors declare that the research was conducted in the absence of any commercial or financial relationships that could be construed as a potential conflict of interest.

## Publisher’s Note

All claims expressed in this article are solely those of the authors and do not necessarily represent those of their affiliated organizations, or those of the publisher, the editors and the reviewers. Any product that may be evaluated in this article, or claim that may be made by its manufacturer, is not guaranteed or endorsed by the publisher.

## Abbreviations

GEO, Gene Expression Omnibus; DEGs, differentially expressed genes; PPI, protein–protein interaction, GO, Gene Ontology, KEGG, Kyoto Encyclopedia of Genes and Genomes, ELISA, enzyme-linked immunosorbent assay; ROC, receiver operating characteristic curves; AUROC, area under the ROC curves; PPV, positive predictive value; NPV, negative predictive value; PLR, positive likelihood ratio; NLR, negative likelihood ratio.
